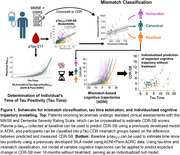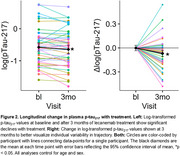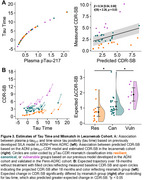# Personalized Monitoring of Response to Anti‐Amyloid Therapy in a Real‐World Cohort using Clinical Assessment and Plasma Biomarkers

**DOI:** 10.1002/alz70861_108800

**Published:** 2025-12-23

**Authors:** Christopher A Brown, Katheryn A. Q. Cousins, Magdalena Korecka, Divij Mathew, Valerie Humphreys, Danielle Hing, Laura Schankel, Alice Chen‐Plotkin, Corey T. McMillan, Eddie B. Lee, Dawn Mechanic‐Hamilton, Leslie M. Shaw, David A. Wolk

**Affiliations:** ^1^ University of Pennsylvania, Philadelphia, PA USA; ^2^ Perelman School of Medicine, University of Pennsylvania, Department of Pathology and Laboratory Medicine, Philadelphia, PA USA; ^3^ Department of Pathology & Laboratory Medicine, Perelman School of Medicine, University of Pennsylvania, Philadelphia, PA USA

## Abstract

**Background:**

Blood‐based biomarkers may be a cost‐effective, accessible method for monitoring response to anti‐amyloid therapy (AAT). While plasma biomarkers of Alzheimer’s disease pathology correlate with amyloid and tau measures on PET and change with AAT in clinical trials, data from real‐world cohorts receiving AAT is limited. Moreover, potential uses beyond treatment eligibility and longitudinal change have not been evaluated. Here we demonstrate the potential clinical utility of plasma Alzheimer’s disease biomarkers in a cohort receiving AAT at the University of Pennsylvania’s Penn Memory Center (PMC).

**Methods:**

43 individuals receiving lecanemab at the PMC had plasma collected at baseline and every 4 weeks after starting AAT. Samples were collected at infusion visits prior to drug infusion, then processed, stored, and analyzed using standardized ADRC protocols. Baseline and 2‐3 month follow‐up measures of *p* ‐tau_217_ were obtained on the Fujirebio Lumipulse platform. Linear‐mixed models evaluated change over time after log‐transforming *p* ‐tau_217_ values. Next, we applied previously developed models from the ADNI+Penn ADRC cohorts to obtain SILA‐estimated time from tau positivity (“Tau Time”) and *p* ‐tau_217_‐CDR‐SB mismatch classification into canonical, resilient, or vulnerable groups (Figure 1). As CDR‐SB is not collected in routine clinical care, we used a crosswalk from MMSE and Dementia Severity Rating Scale based on Penn ADRC data to calculate an estimated CDR‐SB in this dataset. Finally, Tau Time and Mismatch Classification were used to model expected cognitive trajectory over 18 months for the cohort.

**Results:**

Plasma *p* ‐tau_217_ was lower after 3 months of treatment compared to baseline (*β* = ‐0.07 [‐0.12, ‐0.01], *t*(82) = ‐2.33, *p* = 0.018), although there was considerable heterogeneity in change across individuals (Figure 2). The average Tau Time was 3.77 ± 4.64 years, and 51% of individuals were classified as canonical, 32% as resilient, and 17% as vulnerable (Figure 3a). Finally, the expected change in CDR‐SB over 18 months was 1.12 ± 0.72 overall but differed significantly by mismatch group (Figure 3, *t*(37) > 9.8, *p* < 0.001).

**Conclusions:**

Plasma *p* ‐tau_217_ and brief clinical assessments can be used to provide individualized monitoring, and potentially, prediction of treatment response to AAT in real‐world settings.